# NEGATIVE EPISTASIS BETWEEN α^+^ THALASSAEMIA AND SICKLE CELL TRAIT CAN EXPLAIN INTERPOPULATION VARIATION IN SOUTH ASIA

**DOI:** 10.1111/j.1558-5646.2011.01408.x

**Published:** 2011-12

**Authors:** Bridget S Penman, Saman Habib, Kanika Kanchan, Sunetra Gupta, A Read

**Affiliations:** 1 Department of Zoology, University of OxfordSouth Parks Road Oxford, OX1 3PS, United Kingdom; 3 Central Drug Research InstituteLucknow, India

**Keywords:** Epistasis, human evolution, malaria, population genetics, sickle cell, South Asia

## Abstract

Recent studies in Kenya and Ghana have shown that individuals who inherit two malaria-protective genetic disorders of haemoglobin—α^+^ thalassaemia and sickle cell trait—experience a much lower level of malaria protection than those who inherit sickle cell trait alone. We have previously demonstrated that this can limit the frequency of α^+^ thalassaemia in a population in which sickle cell is present, which may account for the frequency of α^+^ thalassaemia in sub-Saharan Africa not exceeding 50%. Here we consider the relationship between α^+^ thalassaemia and sickle cell in South Asian populations, and show that very high levels of α^+^ thalassaemia combined with varying levels of malaria selection can explain why sickle cell has penetrated certain South Asian populations but not others.

It is widely accepted that the high frequencies of genetic blood disorders (haemoglobinopathies) seen in almost all old-world malarious regions are the result of malaria selection (Haldane 1949; Flint et al. 1998). The mutation responsible for sickle cell anaemia (β^S^) serves as a canonical example: the heterozygous condition (sickle cell trait) offers close to 90% protection against severe *Plasmodium falciparum* syndromes (Allison 1964; Hill et al. 1991; Williams et al. 2005a); the homozygous condition causes lethal sickle cell disease. The inactivation of one of the pair of alpha globin genes on chromosome 16 (α^+^ thalassaemia) also offers substantial malaria protection (Williams et al. 2005b) but only causes a mild blood disorder in its homozygous state.

Recent studies have revealed an additional dimension to the relationship between β^S^ and α^+^. A cohort study of 2104 children living in Kilifi, Kenya (Williams et al. 2005c) found that malaria protection was reduced to only 10% in children who inherited both sickle cell trait and homozygous α^+^. May et al. (2007) were able to extend this observation with their finding that Ghanaian children with sickle cell trait and heterozygous α^+^ thalassaemia enjoy a lower degree of malaria protection than children with sickle cell trait alone (the odds ratio for severe malaria disease was 0.06 for sickle cell trait alone, increasing to 0.12 in the presence of α^+^ thalassaemia). Because sickle cell trait is caused by a mutation in beta globin, and α^+^ thalassaemia by a mutation in alpha globin, this is a clear example of epistasis (here defined as the presence of a particular allele at one locus affecting the phenotypic outcome of an allele at a second locus).

The mutation responsible for α^+^ thalassaemia is found in nearly all old-world malarious regions (Flint et al. 1998; Weatherall and Clegg 2001a). However, its frequency in sub-Saharan Africa does not exceed 50%, despite the intense malaria selection present and the relatively benign nature of homozygous α^+^. We know that α^+^ is capable of reaching higher population frequencies elsewhere in the world: in Melanasia it can climb to 68% (Flint et al. 1986). In Williams et al. (2005c) we used a mathematical model to demonstrate that a cancellation of malaria protection when β^S^ is inherited alongside α^+^ could account for why α^+^ frequencies have been capped in sub-Saharan Africa. In Melanasia, a lack of β^S^ has allowed α^+^ to climb higher.

Negative epistasis between alpha thalassaemia and sickle cell trait is not the only example of epistasis among the malaria-protective haemoglobinopathies. Alpha thalassaemia can interact with beta thalassaemia to result in a milder clinical phenotype than would have been seen with beta thalassaemia alone (Weatherall and Clegg 2001b). In a separate modelling exercise (Penman et al. 2009), we considered both positive and negative epistatic interactions together in the context of Mediterranean populations, and argued that positive epistasis in particular could have helped a combination of alpha and beta thalassaemia to keep the highly malaria-protective β^S^ out of much of the Mediterranean region. This led us to the assertion that different patterns of malaria-protective haemoglobinopathies in different populations may be partly maintained by interactions among the genes themselves.

In the Middle East, where sickle cell and α^+^ thalassaemia coexist, the pattern does not appear to be too different from that seen in sub-Saharan Africa. In Saudi Arabia, for example, the highest reported frequency of α^+^ thalassaemia is 0.55 (El-Hazmi and Warsy 1999). In South Asia, the pattern is much more variable. Table 1 summarizes South Asian data from tribal populations from three locations: Orissa (India); Andhra Pradesh (India), and the Terai region of India and Nepal, obtained from the literature and from sequencing work first reported in this paper. These represent populations living in malarious regions for which data on both sickle cell and alpha thalassaemia are available.

**Table 1:** α^+^ and β^S^ frequencies in South Asian populations This table has been compiled from the literature and from some extra sequencing done in preparation for this article. We included only estimates from the literature where the sample size was at least 15, so three of the tribal groups studied by Fodde et al. in their 1991 paper have been left out (the Kolam, the Kotiya, and the Nooka Dora). We also left out the Konda Kammari (from the same paper), because we could find no source for a β^S^ frequency in that group. α^+^ refers to any mutation that eliminates alpha globin production from one of the two alpha globin genes on chromosome 16. Deletions that eliminate alpha globin production from both genes exist (these are usually referred to as α^0^ deletions), but were not reported in any of these specific populations. The vast majority of α^+^ worldwide is caused by either the –α^3.7^ deletion (a result of unequal crossing over between two homologous sections of the chromosome which are 3.7 kb apart) or the –α^4.2^ deletion (a result of unequal crossing over between two homologous sections of the chromosome which are 4.2 kb apart). The 3.7 deletion can be categorized into types I, II, and III depending upon where in the homologous stretch of DNA the crossover occurred. Globally, type I is the most common and type III the rarest. The “other” column in the table notes unusual nondeletional alpha thalassaemic variants such as Haemoglobin Koya Dora (HbKD) and Hb Rampa, or other abnormalities such as the triplication of the alpha globin gene (the other product of unequal crossing over). When an α^+^ and a β^S^ frequency estimate appear in the same row, they were estimated from the same population in the same study. In all other cases, we have had to resort to different studies of the same ethnic group in the same area

		Frequency of α^+^						
Location	Tribal group	–α^3.7^ deletion	–α^4.2^ deletion	Others	Total	Frequency of β^S^	Sources	Number typed
Sundargarh district of Orissa	Munda	0.5			unknown (4.2 frequency yet to be established)	0; 0.016	This article (see Supporting information for genotyping methods) Balgir et al. (2006a) for second β^S^ estimate	44 (this article); 96 (Balgir et al. 2006a)
	Oraon	0.625				0; 0	This article, Balgir et al. (2006a)	36 (this article); 104 (Balgir et al. 2006a)
Central Terai (Nepal)	Tharu	0.83 (type I)	0	None noted	0.83	0	Modiano et al. 1991	18 (α^+^); 124 (β^S^)
Western Terai (Nepal)	Tharu	0.67 (type I), 0.05 (type II)	0	None noted	0.72	0.05	Modiano et al. 1991	18 (α^+^); 185 (β^S^)
Western Terai (India)	Tharu	0.94				0.1	Sinha et al. 2009 (α^+^), this article (β^S^)	53
Andhra Pradesh (AP)	Koya Dora	0.26 (type I) 0.1 (type II)	0.32;	0.12 (HbKD)	0.8	0.12	Fodde et al. 1988;	25
		0.3 (type I) 0.07 (type II)	0.33	0.07 (HbKD); 0.07 (Hb Rampa)	0.77		Fodde et al. 1991	30
						0.088	Nayudu 1990	452
						0.0673	Babu et al. 2002	1099
	Valmiki	0.26 (type I)	0.08	None noted	0.46		Fodde et al. 1991	50
		0.12 (type II)				0.172	Nayudu 1990	553
						0.1216	Babu et al. 2002	950
	Konda Dora	0.18 (type I)	0.32	0.05	0.55		Fodde et al. 1991	22
				(HbKD)		0.0629	Babu et al. 2002	668
	Konda Reddi/Konda Reddy	0.531 (type I)	0	0.0625 (alpha globin triplication)	0.531	0.03	Fodde et al. 1988;	16
		0.35 (type I)	0		0.35		Fodde et al. 1991	17
						0.0696	Nayudu 1990	632
						0.0635	Babu et al. 2002	724
	Bhaghatha/Bagatha	0.44 (type I)	0.26	None noted	0.6		Fodde et al. 1991	27
						0.0618	Babu et al. 2002	283

As shown in Table 1, widely varying frequencies of both α^+^ and β^S^ may be observed in these areas, but it is clear that while all groups possess high frequencies of alpha thalassaemia, not all groups possess sickle cell. The contrast is particularly striking for the central and Western Terai Tharu populations: Tharus in the Western Terai region in Nepal have both sickle cell (0.05) and a high frequency of α^+^ thalassaemia (0.72), but Central Terai Tharus have an α^+^ thal frequency of 0.83 and no sickle cell at all (Modiano et al. 1991). Indian Tharus from the Terai region of Uttar Pradesh have near-fixation of α^+^, at a frequency of 0.94 (Sinha et al. 2009) as well as sickle cell. In this article, we extend the modelling work carried out in Williams et al. (2005c) and Penman et al. (2009) to ask whether these data from South Asia can be reconciled with the negative epistasis between α^+^ thalassaemia and sickle cell documented in Africa.

## Methods

To explore the population genetic consequences of negative epistasis between α^+^ thalassaemia and sickle cell trait, we considered a population containing nine possible genotypes constructed from four possible gametic types: αβ, α^+^β, αβ^S^, and α^+^β^S^.

α^+^β/αβ^S^ is equivalent to αβ/α^+^β^S^. The frequency of genotype *i* is given by *y_i_*.

The rate of change of frequency of each genotype with time was given by:

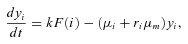
where

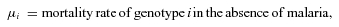


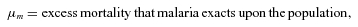


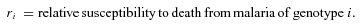


The term *k* gives the total birth rate, and was calculated so as to keep the total population size constant (see Supporting information).

*F*(*i*) apportions the total birth rate into different genotypes according to the frequencies of their possible constituent gametypic types following simple rules of panmixia (see Supporting information). Alpha and beta globin are encoded on chromosomes 16 and 11, respectively, so we have modeled them as being completely unlinked.

We investigated our model's behavior by carrying out many numerical simulations, systematically sampling parameter space to ensure that we had found all possible stable outcomes.

The dynamical framework presented here could be reframed into a more conventional population genetic model by calculating a life expectancy for each genotype (the inverse of its total mortality rate) and taking the ratio of the life expectancy of a given genotype to the longest life expectancy in the population as the relative fitness of that genotype. We chose to use a dynamical approach because the two mortality rates assigned to each genotype make it obvious what we have assumed about blood disorder severity; malaria protection and epistasis for each genotype, rather than subsuming both into a single fitness estimate. This type of dynamical approach has been employed in previous studies of malaria resistance (Gupta and Hill 1995; Ruwende et al. 1995).

The mortality rates used in this article are intended to demonstrate the range of possible model behaviors rather than provide a specific historical recreation. As can be seen in Table S1, we chose 0.03 years^−1^ as the mortality rate of normal individuals in the absence of death from malaria, implying an average life expectancy of 33 years. This seems reasonable, but is an arbitrary baseline—what matters are the relative values of the mortality rates assigned to each genotype. If μ_*i*_= 0.03 and μ_*m*_= 0.01, this implies that malaria is responsible for 25% of the total mortality of wild-type individuals in that population.

## Results and Discussion

**Figure 1:**
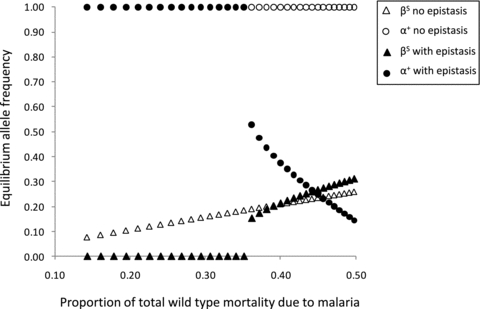
This figure shows how the equilibrium frequencies of α^+^ and β^S^ change with differing levels of malaria selection, with and without negative epistasis. These equilibrium frequencies are obtained after 150,000 years; α^+^ and β^S^ both started out at a frequency of 0.001 in the population. The blood disorder mortality rates and relative susceptibility to death from malaria for each genotype are the unbracketed figures given in Table S1 (figures in italics were used in the “no epistasis” scenario).

Figure 1 demonstrates that the inclusion of negative epistasis has a dramatic impact on the equilibrium frequencies of α^+^ and β^S^ (as already discussed in Williams et al. 2005c). In the absence of epistasis, α^+^ always reaches fixation in the population alongside β^S^; when epistasis is included, antagonism between α^+^ and β^S^ can limit the former's frequency. However, under negative epistasis the system is highly sensitive to the level of malaria selection. At lower levels of malaria selection, α^+^ can become fixed in the population to the complete exclusion of β^S^; the coexistence of α^+^ and β^S^ is only possible above a certain selection threshold.

**Figure 2:**
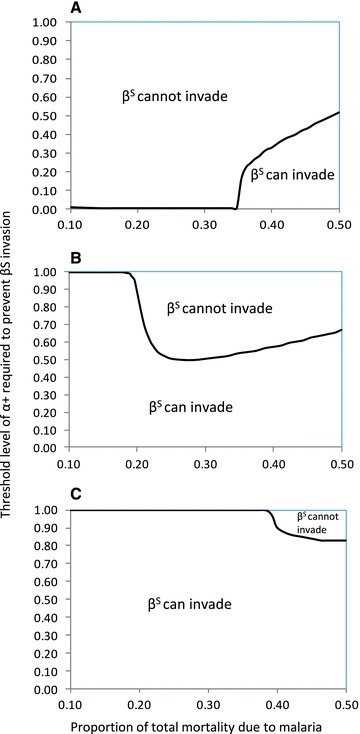
The balance of power between α^+^ and β^S^. Panels (A-C) illustrate the frequency of α^+^ required to prevent sickle cell successfully invading the population, where negative epistasis is present. In panel (A), homozygous α^+^ thalassaemia carries no cost (mortality rate = 0.03 years^−1^). In panel (B), it has been assigned a mortality rate of 0.031 years^−1^, and in panel (C) it has been assigned a mortality rate of 0.032 years^−1^. All other mortality rates are as in Table S1, and include negative epistasis. β^S^ is given an initial frequency of 0.001 in all cases, and “prevention of invasion” is defined as β^S^ being at a frequency below 0.00005 after 50000 years.

Figure 2 considers the possibility of pre-existing high frequencies of α^+^ acting against β^S^. Clearly, even under strong malaria selection, a high frequency of α^+^ can stop β^S^ from invading the population. In Figure 2 we also consider the effect of introducing a cost to the α^+^α^+^ genotype. Nowadays, the mild anaemia associated with α^+^α^+^ is not regarded as a significant health concern, but historically even this mild anaemia may have led to a small increase in mortality (e.g., during childbirth), so a small blood disorder related cost seems plausible. Unsurprisingly, the higher the cost of homozygosity for α^+^, the harder it is for α^+^ to keep β^S^ out of a population. Figure S1 illustrates how this cost affects the equilibrium frequencies of α^+^ and β^S^.

The key observation from Figures 1 and 2 is that, in the presence of negative epstasis, malaria selection has a nonlinear effect on allele frequencies of α^+^ and β^S^. As shown in Figure 2, a small change in malaria selection pressure can move a population from a scenario where α^+^ excludes β^S^ to one where β^S^ keeps α^+^ in check.

As discussed in the Introduction, and detailed in Table 1, β^S^ coexists with a high frequency of α^+^ thalassaemia in the Tharus of the Western Terai. However, Central Terai Tharus possess no β^S^, just α^+^ thalassaemia alone. Similarly, the Munda tribe of Orissa possesses sickle cell, whereas the Oraon do not—but both have high frequencies of α^+^. Figure 2 illustrates that a change in malaria selection pressure could allow β^S^ to invade an α^+^ rich population where previously it was excluded. Negative epistasis between α^+^ and β^S^ combined with varying malaria selection thus offers a potential explanation for the heterogeneity in sickle cell's distribution among the Tharu.

**Figure 3:**
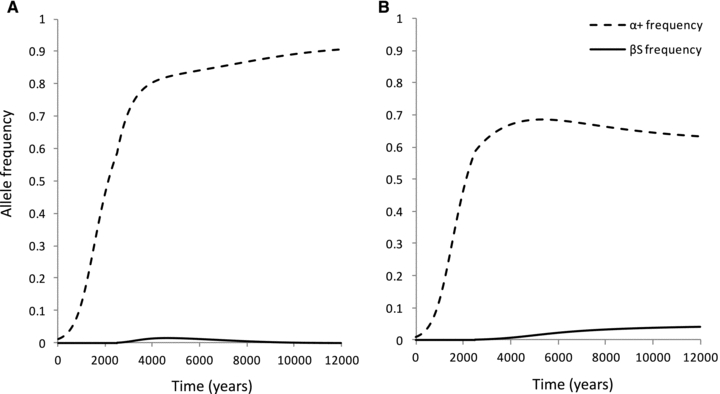
The behavior of α^+^ and β^S^, with epistasis and varying malaria selection levels. Panels (A) and (B) indicate how the equilibrium frequencies of α^+^ and β^S^ change over time. In both panels, the initial frequency of α^+^ was 0.01 at time 0 and malaria was responsible for 19% of the wild-type mortality over the first 2500 years. After 2500 years, β^S^ was introduced at a frequency of 0.001, and the levels of malaria selection changed as follows: in panel (A) malaria became responsible for 27% of total wild-type mortality, and in panel (B) malaria became responsible for 14% of the total wild-type mortality. In these scenarios, we assumed there was a slight cost to the α^+^α^+^ phenotype (it was assigned a mortality rate of 0.031 years^−1^ compared to the wild type 0.03 years^−1^).

Figure 3 provides a time series to illustrate this point. Panels (a) and (b) illustrate two populations that are subject to a high level of malaria selection, and accumulate a high frequency of α^+^. After 2500 years, the β^S^ allele arrives in both populations. In population (a), this coincides with an increase in the level of malaria selection, and β^S^ is kept out. In population (b), this coincides with a drop in the level of malaria selection and β^S^ is allowed in. The fact that an increase in malaria selection keeps β^S^ out is related to the properties we assigned the α^+^ allele in this particular example: as can be seen from Figure 2, there are some regions where an upwards shift in malaria selection will act against β^S^ invasion, and others where an upwards shift in malaria selection will favor β^S^ invasion.

The very high frequencies of alpha thalassaemia seen alongside sickle cell in the tribal groups of Andhra Pradesh present a conundrum, in that there is no evidence for sickle cell being excluded from any population, and the sickle cell frequencies observed are very high (Table 1). Under the negative epistasis model, we have to explain high frequencies of alpha thalassaemia coexisting with high frequencies of sickle cell as being far from equilibrium: this could be due to rapid changes in malaria selection pressure, or the effects of population admixture. In the specific case of the Koya Dora of AP, it might be significant that 10% of alpha thalassaemia in this population is due to a unique mutant of the normal α chain termination codon, dubbed HbKD (De Jong et al. 1975). This mutation leads to an elongation of the alpha globin subunit, similar to that caused by Haemoglobin Constant Spring (Weatherall and Clegg 2001b), and it is conceivable that this elongated alpha globin interacts with sickle beta globin in a way that avoids negative epistasis.

The majority of alpha thalassaemia in India, however, is caused by the same types of deletion as are found in Africa. Unknown effects of HbKD aside, the most likely mechanism behind the negative epistasis observed in Kenya ought to apply equally in India. All sickle haemoglobin is formed of 2 alpha globin and 2 mutated sickle beta globin subunits. The electrostatic properties of the globin subunits mean that alpha globin partners preferentially with normal beta globin over sickle beta globin (Bunn 1987). In erythrocytes with both sickle cell trait and α^+^ thalassaemia, there will be a limited supply of alpha globin; thus, a red blood cell with sickle cell trait and alpha thalassaemia will have a lower intracellular concentration of sickle haemoglobin than a red blood cell with sickle cell trait alone. Williams proposes that the malaria-protective properties of sickle cell trait must rely upon the intracellular concentration of sickle haemoglobin—so accounting for the loss of malaria protection in sickle cell-α^+^ thalassaemic erythrocytes.

The diversity of different mutations present in populations can sometimes be used to gauge the degree of population admixture, or trace migration events that have occurred. In the case of sickle cell, almost all sickle cell in India occurs on the same beta globin haplotype, indicating a spread from a common origin (Flint et al. 1998). Flint et al. note in their review that the mutation may have arisen in or been imported into a single Indian population, which then became dispersed following invasions from the North at some point during the last 5000 years. This could certainly account for some of the heterogeneity in the distribution of sickle cell in South Asia, but cannot explain why some Tharus have it but others do not.

In terms of alpha thalassaemia: table one makes clear that two types of deletion are responsible for most alpha thalassaemia in these populations (–α^3.7^ and –α^4.2^). The –α^3.7^ and –α^4.2^ deletions arise through unequal recombination; are responsible for most alpha thalassaemia worldwide (Weatherall and Clegg 2001b), and have indistinguishable phenotypic effects (Williams et al. 1996). The predominance of the –α^3.7^ deletion in the Tharu may indicate especially strong malaria selection (leading to the rapid spread of the first deletion that occurred), or perhaps a lack of population admixture—but further modelling work is necessary to investigate exactly how different recombination rates, mutation rates, selection levels, and migration between populations interact to determine alpha thalassaemic diversity.

As noted in the Introduction, we have already argued that a combination of alpha and beta thalassaemia may be helping to exclude sickle cell from much of the Mediterranean (Penman et al. 2009). Alpha thalassaemia frequencies in the Mediterranean are lower than in the populations considered here, and we did not consider exclusion by alpha thalassaemia alone a robust explanation for the rarity of sickle cell in Greece and Cyprus, but positive epistasis between alpha and beta thalassaemia could have assisted beta thalassaemia in outcompeting sickle cell. In addition to beta thalassaemia in the Mediterranean, sickle cell competes with beta thalassaemia and the structural variant haemoglobin C (HbC) in West Africa (Livingstone 1976; Hedrick 2004; Modiano et al. 2007), and mutual exclusion appears to occur between β^S^ and haemoglobin E (HbE), in Asia (Weatherall and Clegg 2001a, see Figure 2). Could competition with another beta globin variant be responsible for some of the patchiness in sickle cell's distribution in India and Nepal?

Modiano et al. (1991) observed a low frequency (0.02) of beta thalassaemia in Central Terai Tharus. It is conceivable that this small amount of beta thalassaemia acts synergistically with alpha thalassaemia to exclude sickle cell from this population, as we suggested in the context of the Mediterranean in Penman et al. (2009), but it is just as possible that alpha thalassaemia kept sickle cell out, allowing beta thalassaemia to appear later. Unfortunately, Modiano et al. (1991) did not record the frequency of beta thalassaemia in the Western Terai Tharu population—further studies of Tharu populations may help to clarify this point.

Varying levels of beta thalassaemia occur in the tribal populations of Orissa (Balgir 2006a,b). Of the populations we consider in Table 1, the Munda were reported as having a beta thalassaemia allele frequency of 0.026, and the Oraon a frequency of 0.009. Balgir observed a nonsignificant negative correlation between β^S^ and beta thalassaemia frequencies. It seems likely that β^S^ and beta thalassaemia compete in this region, but the relationship between them is far from straightforward. The results we present here suggest that to understand heterogeneity in the distribution of β^S^ fully, we must consider alpha as well as beta thalassaemia.

Prior to Williams et al. (2005c), a different form of epistatic interaction between α^+^ and β^S^ had been suggested: namely that α-thalassaemia may be able to ameliorate some of the adverse symptoms of sickle cell anaemia. The proposed mechanism for this effect once again hinges on a limited pool of α-globin leading to a lower concentration of sickle haemoglobin in red blood cells. In the case of sickle cell anaemia, there is only sickle beta globin available—no normal beta globin—but some of the alpha globin binds to the very small amount of delta globin available in erythrocytes, and when alpha globin is limited this becomes more important. Although it is clear that alpha thalassaemia can change the hematological profile of sickle cell disease, it is less clear that this has any beneficial effect in terms of increased survival (see Weatherall and Clegg 2001b, especially table 11.8 on p. 523). Nevertheless, in the Supporting information, we investigate how such an effect might interact with negative epistasis canceling malaria protection for sickle heterozygotes. It does not seem to alter the overall pattern (Fig. S2).

The strikingly different haemoglobinopathy patterns observed in different world regions each represent an alternative evolutionary answer to the problem of malaria. It is starting to become clear that the success or failure of particular beta globin mutations may be affected by the presence or absence of alpha globin mutations, and vice versa. Here we have presented a detailed exploration of the range of behaviors allowed when α^+^ and β^S^ interact via negative epistasis. Small changes in malaria selection pressure can dramatically alter the prospects of sickle cell invading a population. Such effects could account for some of the variation in sickle cell frequency among specific populations in India and Nepal.

## Supporting Information

The following supporting information is available for this article:

**Figure S1.** The effect of varying the severity of α^+^ thalassaemia on the equilibrium frequencies of α^+^ and β^S^.

**Figure S2.** Allowing α^+^ thalassaemia to alleviate sickle cell anaemia.

**Table S1.** Mortality rates used to generate the figures.

## References

[b1] Allison AC (1964). Polymorphism and natural selection in human populations. Cold Spring Harb. Symp. Quant. Biol.

[b2] Babu BV, Krishna Leela BL, Kusuma YS (2002). Sickle cell disease among tribes of Andhra Pradesh and Orissa States, India. Anthropologischer Anzeiger.

[b3] Balgir RS (2006a). Do tribal communities show an inverse relationship between sickle cell disorders and glucose-6-phosphate dehydrogenase deficiency in malaria endemic areas of central-eastern India?. HOMO J. Comp. Hum. Biol.

[b4] Balgir RS (2006b). Genetic heterogeneity of population structure in 15 major scheduled tribes in central-eastern India: a study of immuno-hematological disorders. Ind. J. Hum. Genet.

[b5] Bunn HF (1987). Subunit assembly of hemoglobin: an important determinant of hematologic phenotype. Blood.

[b6] De Jong WW, Meera Khan P, Bernini LF (1975). Hemoglobin Koya Dora: high frequency of a chain termination mutant. Am. J. Hum. Genet.

[b7] El-Hazmi MAF, Warsy AS (1999). Appraisal of sickle-cell and thalassaemia genes in Saudi Arabia. Eastern Mediterranean Health J.

[b8] Flint J, Harding RM, Boyce AJ, Clegg JB (1998). The population genetics of the haemoglobinopathies. Baillieres Clin. Heamatol.

[b9] Flint J, Hill AVS, Bowden DK (1986). High frequencies of α-thalassaemia are the result of natural selection by malaria. Nature.

[b10] Fodde R, Harteveld CL, Losekoot M, Giordano PC, Meera Khan P, Nayudu NVS, Bernini LF (1991). Multiple recombination events are responsible for the heterogeneity of alpha-thalassemia haplotypes among the forest tribes of Andhra Pradesh, India. Ann. Hum. Genet.

[b11] Fodde R, Losekoot M, Van DB, Oldenburg M, Rashida N, Schreuder A, Wijnen JT, Giordano PC, Nayudu NVS, Meera Khan P, Bernini LF (1988). Prevalence and molecular heterogeneity of alpha+ thalassemia in two tribal populations from Andhra Pradesh, India. Hum. Genet.

[b12] Gupta S, Hill AVS (1995). Dynamic interactions in malaria: host heterogeneity meets parasite polymorphism. Proc. R. Soc. Lond. B.

[b13] Haldane JBS (1949). Disease and evolution. Ric. Sci. Suppl: A.

[b14] Hedrick P (2004). Estimation of relative fitnesses from relative risk data and the predicted future of haemoglobin alleles S and C. J. Evol. Biol.

[b15] Hill AVS, Allsopp CEM, Kwiatkowski D, Anstey NM, Twumasi P, Rowe PA, Bennett S, Brewster D, McMichael AJ, Greenwood BM (1991). Common West African HLA antigens are associated with protection from severe malaria. Nature.

[b16] Livingstone FB (1976). Hemoglobin history in West Africa. Human Biol.

[b17] May J, Evans JA, Timmann C, Ehmen C, Busch W, Thye T, Agbenyega T, Horstmann RD (2007). Hemoglobin variants and disease manifestations in severe falciparum malaria. J. Am. Med. Assoc.

[b18] Modiano D, Bancone G, Ciminelli BM, Pompei F, Blot I, Simpore J, Modiano G (2007). Haemoglobin S and haemoglobin C: ‘quick but costly’ versus ‘slow but gratis’ genetic adaptations to *Plasmodium falciparum* malaria. Hum. Mol. Genet.

[b19] Modiano G, Morpurgo G, Terrenato L, Novelletto A, Di Rienzo A, Colombo B, Purpura M, Mariani M, Santachiara-Benerecetti S, Brega A (1991). Protection against malaria morbidity: near-fixation of the α-thalassemia gene in a Nepalese population. Am. J. Hum. Genet.

[b20] Nayudu NV (1990). Field survey for sickle cell disease in the tribal population of East Godavari District, Andhra Pradesh. J. Association of Physicians of India.

[b21] Penman BS, Pybus OG, Weatherall DJ, Gupta S (2009). Epistatic interactions between genetic disorders of hemoglobin can explain why the sickle-cell gene is uncommon in the Mediterranean. Proc. Natl. Acad. Sci. USA.

[b22] Ruwende C, Khoo SC, Snow RW, Yates SNR, Kwiatkowski D, Gupta S, Warn P, Allsopp CEM, Gilbert SC, Peschu N (1995). Natural selection of hemi- and heterozygotes for G6PD deficiency in Africa by resistance to severe malaria. Nature.

[b23] Sinha S, Arya V, Agarwal S, Habib S (2009). Genetic differentiation of populations residing in areas of high malaria endemicity in India. J. Genet.

[b24] Weatherall DJ, Clegg JB (2001a). Inherited haemoglobin disorders: an increasing global health problem. Bull. World Health Organization.

[b25] Weatherall DJ, Clegg JB (2001b). The thalassaemia syndromes.

[b26] Williams TN, Maitland K, Ganczakowski M, Peto TEA, Clegg JB, Weatherall DJ, Bowden DK (1996). Red blood cell phenotypes in the α^+^ thalassaemias from early childhood to maturity. Br. J. Haematol.

[b27] Williams TN, Mwangi TW, Wambua S, Alexander ND, Kortok M, Snow RW, Marsh K (2005a). Sickle cell trait and the risk of plasmodium falciparum malaria and other childhood diseases. J. Infect. Dis.

[b28] Williams TN, Wambua S, Uyoga S, Macharia A, Mwacharo JK, Newton CRJC, Maitland K (2005b). Both heterozygous and homozygous α^+^ thalassemias protect against severe and fatal plasmodium falciparum malaria on the coast of Kenya. Blood.

[b29] Williams TN, Mwangi TW, Wambua S, Peto TEA, Weatherall DJ, Gupta S, Recker M, Penman BS, Uyoga S, Macharia A (2005c). Negative epistasis between the malaria-protective effects of alpha + thalassemia and the sickle cell trait. Nat. Genet.

