# Supplementary material for: NEGATIVE EPISTASIS BETWEEN α+ THALASSAEMIA AND SICKLE CELL TRAIT CAN EXPLAIN INTERPOPULATION VARIATION IN SOUTH ASIA
Source: Evolution. 2011 Dec;65(12):3625–32. doi: 10.1111/j.1558-5646.2011.01408.x (PMC3263337; doi:10.1111/j.1558-5646.2011.01408.x)
Supplement: Supplementary file 1 [file evo0065-3625-SD1.doc]

Supplementary material

Supplementary methods: genotyping

Sickle cell Hb from tribal populations (44 Munda and 36 Oraon individuals) inhabiting the Sundergarh district of the Indian state of Orissa was analyzed by the hemoglobin membrane electrophoresis assay (Kohn, 1969) with 10% samples being confirmed by SNaPshot analysis. All samples from the Tharu tribe inhabiting the low-land Terai region of the state of Uttar Pradesh were genotyped by SNaPshot. For SnaPshot analysis, the fragment of the HbB gene was PCR-amplified from genomic DNA using the primer pair 5’-CTGTGTTCACTAGCAACCTCAAACAGAC-3’ (forward primer) and 5’- GCCCATAACAGCATCAGGAGTGGAC-3’ (reverse primer). The purified PCR product was subjected to single base pair extension thermal cycling using the primer 5’-GACACCATGGTGCATCTGACTCCTG-3’ in the presence of fluorescently-labeled ddNTPs. The reactions were loaded on the ABI 3130xl automated DNA sequencer and the data was analyzed with Gene Mapper v3.5 software (ABI).

The prevalence of the α-thalassemia –α^3.7^ deletion mutation in 63, 44 and 36 members of the Indian Tharu, Munda and Oraon tribes, respectively was determined by gap-PCR (Baysal and Huisman 1994) as described in Sinha *et al*. (2009).

Supplementary methods: the model

The numbers identifying each genotype (1-9) are as follows:

| subscript | *Alpha globin* | *Beta globin* |
| --- | --- | --- |
| 1 | αα | ββ |
| 2 | α^+^α | ββ |
| 3 | α^+^α^+^ | ββ |
| 4 | αα | ββ^S^ |
| 5 | α^+^α | ββ^S^ |
| 6 | α^+^α^+^ | ββ^S^ |
| 7 | αα | β^S^β^S^ |
| 8 | α^+^α | β^S^β^S^ |
| 9 | α^+^α^+^ | β^S^β^S^ |

The birth rate functions F_1_ to F_9_ are as follows:

$$F_{1}=G_{1}^{2}$$

$$F_{2}=2G_{1}G_{2}$$

$$F_{3}=G_{2}^{2}$$

$$F_{4}=2G_{1}G_{3}$$

$$F_{5}=2(G_{1}G_{4}+G_{2}G_{3})$$

$$F_{6}=2G_{2}G_{4}$$

$$F_{7}=G_{3}^{2}$$

$$F_{8}=2G_{3}G_{4}$$

$$F_{9}=G_{4}^{2}$$

These expressions are based on the model given in (Nei, 1965), but we have not included inbreeding.

G_i_ represents the frequency of different possible gametes, and is calculated as follows:

$G_{1}=\frac{y_{1}+0.5(y_{2}+y_{4})+0.25y_{5}}{\sum_{i=1}^{i=6} y_{i}}$ (frequency of gamete α β)

$G_{2}=\frac{y_{3}+0.5(y_{2}+y_{6})+0.25y_{5}}{\sum_{i=1}^{i=6} y_{i}}$ (frequency of gamete α^+^ β)

$G_{3}=\frac{0.5(y_{4})+0.25y_{5}}{\sum_{i=1}^{i=6} y_{i}}$ (frequency of gamete α β^S^)

$G_{4}=\frac{0.5(y_{6})+0.25y_{5}}{\sum_{i=1}^{i=6} y_{i}}$ (frequency of gamete α^+^ β^S^)

These gamete frequencies assume that all β^S^β^S^ genotypes are lethal in early childhood.

Finally, the birthrate *k* was calculated as follows:

$$k=\sum_{i=1}^{i=9} (\mu_{i}+r_{i}\mu_{m})y_{i}$$

The above expressions assume that the β^S^β^S^ genotype is invariably lethal and does not contribute any alleles to the next generation. This version of the model was used throughout the main text, and for figure S1. However, for figure S2 we allowed homozygous α^+^ to alleviate the blood disorder caused by the β^S^β^S^ genotype. In that case we calculated the gamete frequencies as follows:

$G_{1}=\frac{y_{1}+0.5(y_{2}+y_{4})+0.25y_{5}}{\sum_{i=1}^{i=6} y_{i}+y_{9}}$ (frequency of gamete α β)

$G_{2}=\frac{y_{3}+0.5(y_{2}+y_{6})+0.25y_{5}}{\sum_{i=1}^{i=6} y_{i}+y_{9}}$ (frequency of gamete α^+^ β)

$G_{3}=\frac{0.5(y_{4})+0.25y_{5}}{\sum_{i=1}^{i=6} y_{i}+y_{9}}$ (frequency of gamete α β^S^)

$G_{4}=\frac{y_{9}+0.5(y_{6})+0.25y_{5}}{\sum_{i=1}^{i=6} y_{i}+y_{9}}$ (frequency of gamete α^+^ β^S^)

Supplementary results

Figure 1 of the main text illustrates the central result of the modelling exercise originally presented in (Williams et al., 2005). Negative epistasis between alpha thalasssaemia and sickle cell trait (as observed by Williams et al) can lead to the frequency of alpha thalssaemia being limited by the presence of sickle cell– or even to sickle cell being excluded from the population by alpha thalassaemia. How sensitive is this result to different assumptions about the severity of alpha thalassaemia?

Figure S1a reproduces the result given in Figure 1 – we shall regard this as the classic behaviour of the model. If we introduce a slight cost to homozygous α^+^ thalassaemia, we no longer see α^+^ thalassaemia exclude sickle cell at lower levels of malaria selection (figure S1b). Nowadays the mild anaemia associated with the α^+^α^+^ genotype is not regarded as a significant health concern, but historically even this mild anaemia may have led to a small increase in mortality (e.g. during childbirth). We might continue this line of reasoning to suppose that the historic cost of α^+^α^+^ was high enough to limit the frequency of α^+^ to below 50% without needing to invoke negative epistasis, as in figure S1d. However, if that really was the case, why should α^+^ have been able to climb so high in regions such as Papua New Guinea (see Williams *et al*, 2005) ? Effective limitation of α^+^ can be achieved over a range of malaria selection pressures by a combination of negative epistasis and a slight cost to homozygous α^+^ thalassaemia, whilst still allowing α^+^ to reach higher frequencies in the absence of negative epistasis (figure S1b) - robustly accounting for the pattern in sub-Saharan Africa in addition to the contrasting pattern in Papua New Guinea.

As discussed in the main text, there have been some suggestions that α-thalassaemia may be able to ameliorate some of the adverse symptoms of sickle cell anaemia. In figure S2 we investigate the effect of including such an alleviation of the blood disorder. This does not significantly alter the pattern seen.

**Figure S1: The effect of varying the severity of α^+^ thalassaemia on the equilibrium frequencies of α^+^ and β^S^**

In panel (a), the mortality parameters are the unbracketed figures listed in every cell of Table S1. Figures in italics were used in the ‘no epistasis’ scenarios. In panels (b-d) we have reduced the fitness of alpha thalassaemia by increasing the blood disorder associated mortality rate of homozygous α^+^ thalassaemia. In panel (b) this mortality rate was to 0.031 years^-1^, in panel (c) 0.032 years^-1^, in panel (d) 0.035 years^-1^. The initial frequencies of α^+^ and β^S^ were 0.001, and each equilibrium point was obtained by allowing the population to evolve for 150000 years.

**Figure S2: Allowing α^+^ thalassaemia to alleviate sickle cell anaemia.**

The filled circles and triangles repeat the scenario given in panel (a) of figure S1; the two different crosses represent a scenario where everything has been kept the same except the genotype α^+^α^+^ β^S^β^S^ was given a blood disorder mortality rate of 0.035 years^-1^ alongside a susceptibility to malaria of 0.9. Negative epistasis has been included in both of these scenarios.

**Table S1: mortality rates used to generate the figures**

The relative susceptibilities to death from malaria are taken from the Williams study (Williams 2005, see particularly table 2). In order to remove negative epistasis from the scenario, all ββ^S^ genotypes were assumed to enjoy complete protection against death from malaria (figures in italic). The blood disorder associated mortality rates were chosen to examine different possibilities: that homozygous α^+^ thalassaemia carries no anaemia related fitness cost, or that it carries a range of fitness costs (the figures in brackets). In most simulations the β^S^β^S^ genotypes were assumed to be incapable of passing their genes onto the next generation; this was accounted for in the model itself. In one simulation (figure S2) we allowed homozygous α+ thalassaemia to alleviate sickle cell anaemia, and used the mortality rates given in underlined italics.

| **Blood disorder associated mortality rates (µ), years^-1^** | | | |
| --- | --- | --- | --- |
|  | ββ | ββ^S^ | β^S^β^S^ |
| αα | 0.03 | 0.03 | 1 |
| α^+^α | 0.03 | 0.03 | 1 |
| α^+^α^+^ | 0.03 (0.031,0.032,0.035) | 0.03 (0.031,0.032,0.035) | 1 *0.035* |
| **Relative susceptibility to death from malaria (r)** | | | |
|  | ββ | ββ^S^ | β^S^β^S^ |
| αα | 1 | 0 | 0 |
| α^+^α | 0.6 | 0.11, *0* | 0 |
| α^+^α^+^ | 0.54 | 0.9, *0* | 0 *0.9* |

**References:**

Baysal E. and Huisman T. H. J. 1994 Detection of common deletional α-thalassemia-2 determinants by PCR. *Am. J. Hematol*. **46,** 208-213.

Kohn J. (1969) Separation of hemoglobins on cellulose acteate. *J. Clin. Pathol*., **22**: 109.

Nei, M. 1965. Effect of linkage on the genetic load manifested under inbreeding. *Genetics* **51:** 679-688.

Sinha S, Arya V, Agarwal S, Indian Genome Variation Consortium and Habib S (2009) Genetic differentiation of populations residing in areas of high malaria endemicity in India. Journal of Genetics **88**:77-80.

Williams, T.N., Mwangi, T.W., Wambua, S., Peto, T.E.A., Weatherall, D.J., Gupta, S., Recker, M., Penman, B.S., Uyoga, S., Macharia, A., Mwacharo, J.K., Snow, R.W. & Marsh, K. 2005. Negative epistasis between the malaria-protective effects of alpha+ thalassemia and the sickle cell trait. *Nat. Genet.* **37:** 1253-1257.
